# Editorial: Infectious diseases and hematology: diagnosis and management, volume II

**DOI:** 10.3389/fmed.2025.1769806

**Published:** 2026-01-26

**Authors:** Alessandro Perrella, Tomás José González-López

**Affiliations:** 1 UOC Emerging Infectious Disease and at High Contagiousness Infectious Disease Specialist, Department of Azienda Ospedaliera Dei Colli, Cotugno Hospital, Hospital D. Cotugno, Naples, Italy; 2 Department of Hematology, Hospital Universitario de Burgos, Burgos, Spain

**Keywords:** biomarkers, hematology, immunosuppression, infections, transplantation

## Introduction

The interplay between infectious diseases and hematology has long been recognized as one of the most intellectually demanding and clinically volatile landscapes in modern medicine. It is a symbiotic relationship of destruction and defense; hematological malignancies and their requisite therapies dismantle the immune system, inviting infectious agents to invade, while infectious agents themselves can drive hematological pathology, triggering malignancies or mimicking them in confusing diagnostic masquerades.

As we present Volume II of the Research Topic “*Infectious diseases and hematology: diagnosis and management*,” we find ourselves at a distinct historical inflection point. In the wake of a global pandemic and in the midst of a revolution in targeted immunotherapies, the rules of engagement are changing. We are no longer merely managing standard opportunistic infections in standard hosts. We are navigating a new “immunological milieu,” characterized by T-cell exhaustion, B-cell aplasia induced by bispecific antibodies and CAR-T cells, and the persistence of viral pathogens in reservoirs we are only just beginning to map. This editorial serves not only to introduce the excellent contributions contained within this volume but to contextualize them within this rapidly evolving clinical reality.

## The long shadow of COVID-19 and viral persistence

The SARS-CoV-2 pandemic has forced a forced marriage between our two specialties, revealing the profound vulnerability of the hematological patient. While the general population has moved toward a phase of endemicity, the hematological ward remains a frontline. This is starkly illustrated by the contribution from Suzuki et al. regarding persistent COVID-19 in a patient with diffuse large B-cell lymphoma. Their case report is more than an anecdote; it is a warning signal regarding the use of bispecific antibodies (such as epcoritamab) combined with anti-CD20 agents. It demonstrates how modern therapies, while potent against cancer, can render the host an indefinite incubator for viral evolution. This necessitates a paradigm shift: for the hematologist, the “cure” for cancer cannot be divorced from the viral status of the host; for the infectious disease specialist, the definition of “clearance” must be rewritten.

However, the interaction between the virus and the host is bidirectional. While therapies compromise immunity, innate factors may offer protection. Ahmed et al. provide a fascinating exploration into the protective role of natural anti-carbohydrate antibodies against symptomatic SARS-CoV-2. By examining the ABO blood group antigens and their interaction with the virus, this study reminds us that susceptibility is often written into our genetic and serological code long before infection occurs. Furthermore, as we analyze the changing face of the virus itself, Yuan et al. offer critical epidemiological data on the characteristics of non-severe Omicron subvariants. Understanding these subtle shifts in viral pathogenicity is essential for risk stratification in hematology units, where even “mild” variants can trigger severe cascades.

## The transplant frontier: old foes in new scenarios

While novel viruses capture headlines, the “classic” opportunistic pathogens remain the primary drivers of morbidity in transplant medicine. The expansion of haploidentical hematopoietic stem cell transplantation (haplo-HSCT) has democratized access to curative therapy but has also introduced complex immunological hurdles. Feng et al. address this head-on with their retrospective analysis of cytomegalovirus (CMV) infection in aplastic anemia patients undergoing haplo-HSCT. CMV remains the “troll under the bridge” of transplant success. This study is particularly valuable because it moves beyond generalities, identifying specific risk factors in the aplastic anemia cohort. It reinforces the axiom that despite newer antivirals, vigilant surveillance and preemptive strategies remain the bedrock of survival. The nuances of immune reconstitution in the haplo-setting are distinct, and this paper adds a crucial brick to the wall of our understanding.

## Diagnostic precision and prognostic biomarkers

One of the recurring themes of our careers, and indeed of this Research Topic, is the danger of diagnostic ambiguity. In the intersection of ID and Hematology, a misdiagnosis is often fatal. We are presented with a fascinating paradox in the work by Wang et al. regarding HIV screening. We often assume that higher sensitivity is always better. However, this report highlights how fourth-generation assays, while highly sensitive, can paradoxically lead to longer times to confirmatory diagnosis due to discordant results in early seroconversion windows or “grey zone” reactivities. In a hematological setting, where a patient may present with lymphadenopathy that could be lymphoma or acute HIV, such delays are unacceptable. This paper serves as a vital reminder that we must understand the limitations of our tools as intimately as their strengths.

Furthermore, the search for reliable biomarkers continues to dominate research efforts. In the chaotic physiology of the septic ICU patient, distinguishing between reversible inflammation and terminal decline is an art form. Yao et al. investigate the prognostic value of Heat Shock Protein 27 (HSP27) in septic patients. Their findings suggest that HSP27 could serve as a stratification tool for 28-day mortality. In the context of neutropenic sepsis—a daily reality for hematologists—having such biomarkers could eventually help us decide which patients require escalation of therapy and which can be managed more conservatively.

## The masquerade: when infection mimics malignancy (and vice versa)

The clinical acumen of the specialist is tested most severely when diseases do not read the textbook. The volume includes important case reports that highlight these diagnostic chameleons. Du et al. describe a rare presentation of acute promyelocytic leukemia (APL) manifesting as a myeloid sarcoma of the lumbar spine. While primarily a hematological malignancy, such presentations frequently mimic spinal abscesses or tuberculosis (Pott's disease), leading to initial referrals to infectious disease units. Conversely, Zhao et al. reports on severe drug-induced immune hemolytic anemia caused by piperacillin. As infectious disease specialists, piperacillin-tazobactam is one of our workhorses. This case is a sobering reminder of the iatrogenic harm we can inflict; the treatment of the infection became the cause of the hematological crisis. It underscores the necessity of a holistic view: we must treat the infection, but we must also respect the blood.

## Standardization of care: the path forward

As we accumulate individual data points and case reports, the ultimate goal is to synthesize this knowledge into consensus. The “art” of medicine must eventually become the “science” of guidelines. In this regard, the Chinese expert consensus on the application of intravenous immunoglobulin (IVIG), presented by Guo et al., is a monumental contribution to this volume. IVIG is a precious, limited, and expensive resource, frequently used off-label in both infectious and hematological scenarios. By establishing clear, evidence-based consensus for its application in hematological diseases, this document provides a framework for stewardship and efficacy. It is exactly the kind of structured guidance that clinicians on the ground need to make ethical and effective decisions.

In conclusion as we reflect on the diverse array of articles in this second volume, we are struck by the accelerating complexity of our field. The days of treating “the leukemia” and “the pneumonia” as separate entities are over. The modern patient is a complex biological system where the tumor, the immune system, and the microbiome (including the virome) are in a constant, dynamic flux.

This volume does not claim to solve all these problems. Instead, it offers a roadmap. It identifies the new risks of novel therapies, refines our management of established threats (Feng et al.), challenges our diagnostic algorithms (Wang et al.), and pushes for standardization of supportive care (Guo et al.).

We extend our deepest gratitude to the authors for their rigorous research and to the reviewers whose invisible labor upholds the integrity of this journal. It is our hope that this editorial and the Research Topic it introduces will serve as a catalyst for the multidisciplinary collaboration that is no longer an option, but a necessity, for the survival of our patients.

